# The significance of double mandibular osteotomy for surgical treatment of large aneurysm of internal carotid artery above mandibular angle. A case report and literature review

**DOI:** 10.1093/jscr/rjac534

**Published:** 2022-11-23

**Authors:** Ioannis Tilaveridis, Dimitris Tatsis, Gregory Venetis, Georgios Trelopoulos, Anastasios Mylonas

**Affiliations:** Aristotle University of Thessaloniki, Thessaloniki 54124, Greece; Aristotle University of Thessaloniki, Thessaloniki 54124, Greece; General Hospital G. Papanikolaou, Thessaloniki 57010, Greece; Aristotle University of Thessaloniki, Thessaloniki 54124, Greece; General Hospital G. Papanikolaou, Thessaloniki 57010, Greece; Metropolitan Hospital Athens, and Private Practice, Athens 18547, Greece

**Keywords:** aneurysm, surgical approach, double mandibular osteotomy, internal carotid artery, mandibular ‘swing’

## Abstract

The aim of this study is to report a case of an unusual large aneurysm of the internal carotid approached with a doubly mandibular osteotomy in order to prove the significance and the excellent result of the specified surgical procedure because of the cooperation of maxillofacial and vascular surgeons. The double mandibular osteotomy is described, as well as the bypass of the aneurysm and the anastomosis of the peripheral edges of the artery. The double mandibular osteotomy can deliver the internal carotid artery from the bifurcation to its entrance to the skull base through the carotid canal and can offer the opportunity to the vascular surgeon to perform the anastomosis easily, quickly and safely.

## INTRODUCTION

Isolated true aneurysms of the extracranial internal carotid artery (ICA) are rare vascular disorders with its incidence unknown. Only 12 cases can be accounted in the literature; however, other investigators account 0.2–5% of all carotid artery operations [1–3]. The most common site is the bifurcation of the common carotid artery (CCA) and follows the mid- to distal part of the ICA.

Atherosclerosis is the most common causative factor, followed by fibromuscular dysplasia, trauma, or infection [2, 3]. Previous neck dissection, radiotherapy or previous endarterectomy have also been described as risk factors [4, 5].

Treatment of the extracranial ICA aneurysms includes the pharmaceutical use of anticoagulant agents, surgical operation or endovascular stent placement [4–6]. The aneurysm location is a key factor for surgical treatment. According to Blaisdell, aneurysms located below the line connecting the mastoid process with the angle of the mandible can be easily accessed, but lesions above this line are treated with difficulty as they are located into the parapharyngeal space and the ramus of the mandible is a major obstacle [2, 3, 7].

The size of the aneurysm is a crucial factor as it makes the surgical operation more demanding and challenging and more susceptible to cranial nerve damage or dysfunction [1]. Very few cases were larger than 5 cm have been described.

Mandibular subluxation to access the parapharyngeal space has been described, without reference to the morbidity caused by the mandibular function after the subluxation.

The aim of this paper is to present a case of a large (>4 cm) extracranial ICA aneurysm, which was treated with double mandibular osteotomy and transposition of the external carotid surgery (ECA) for anastomosis with the distal part of the ICA near the skull base.

## CASE REPORT

**Figure 1:**
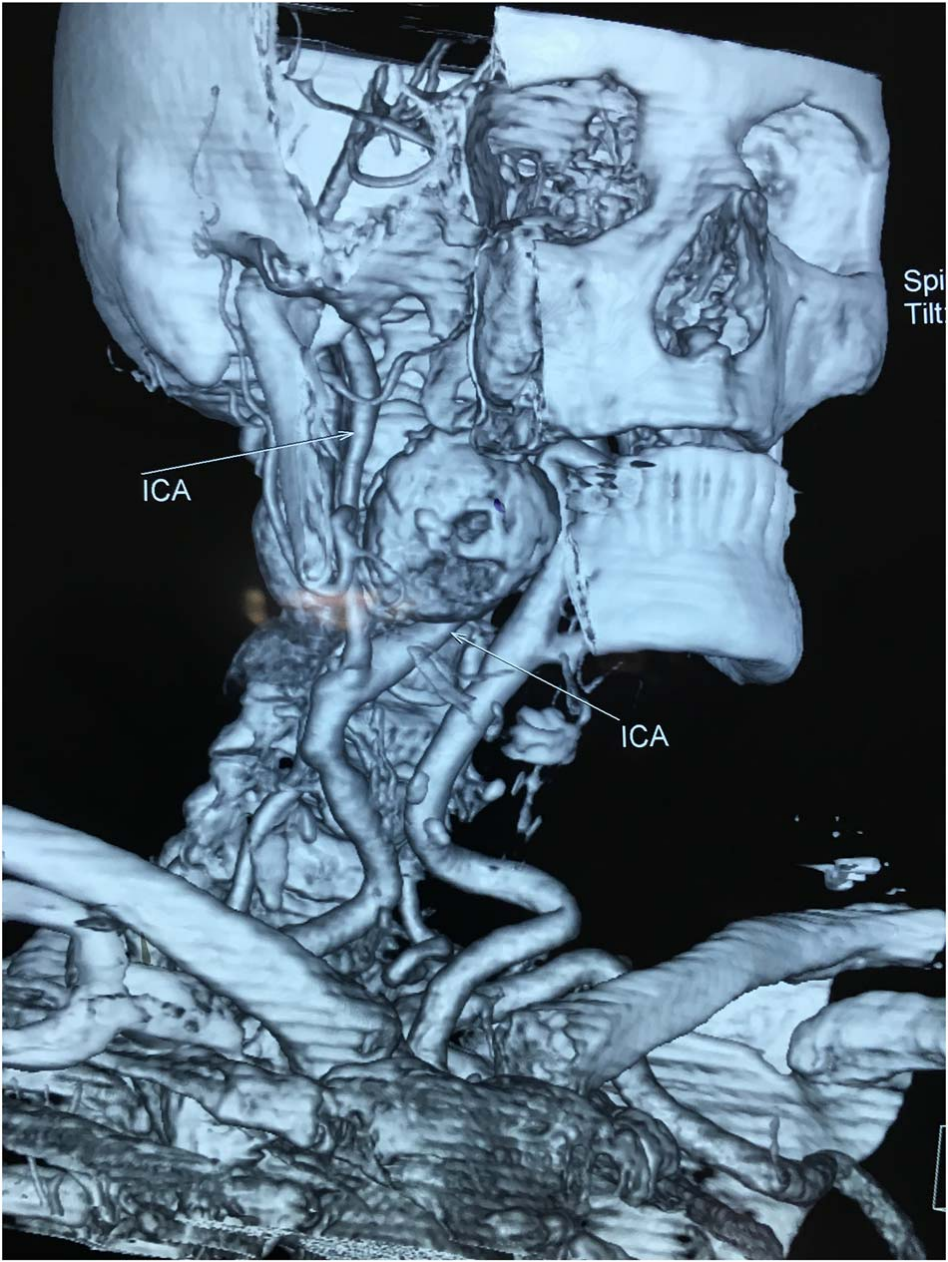
Computed tomography angiography of the carotids with 3D reconstruction.

**Figure 2:**
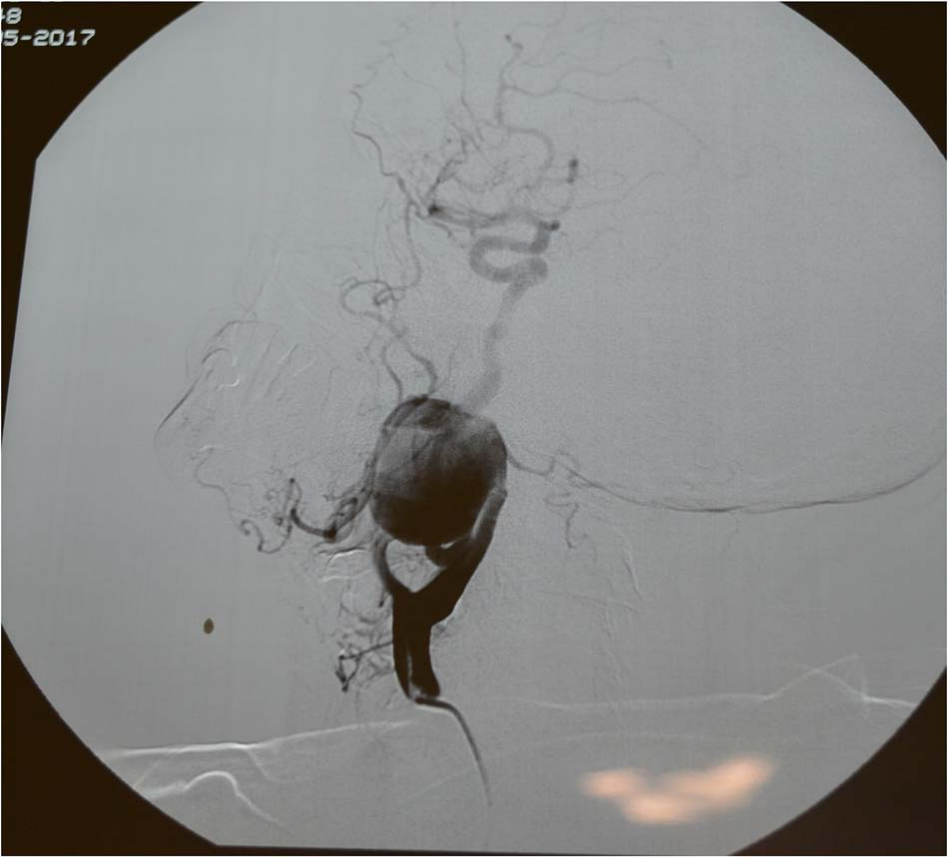
Classical angiography.

A 71-year-old woman was referred to the vascular surgery department of our hospital with an asymptomatic, pulsative, submandibular mass that she had noticed in the right side of her neck for a month and with ‘potato voice’. Her medical history was non-contributory. MRI, 3D-CT (Fig. 1) and conventional angiography (Fig. 2) revealed an aneurysm of the right ICA. The aneurysm was located in the upper third of the artery and its maximal diameter was more than 4 cm, lining to the right parapharyngeal space, whereas the aerodigestive track was limited and dislocated in the height of the aneurysm.

An open surgical treatment was decided as the treatment of choice, because of the aneurysm’s location—over the Blaisdell line—and size. In the preoperative check-up, the simple Matas Test was performed to estimate the danger of possible neurological signs and the back flow intraoperatively.

**Figure 3:**
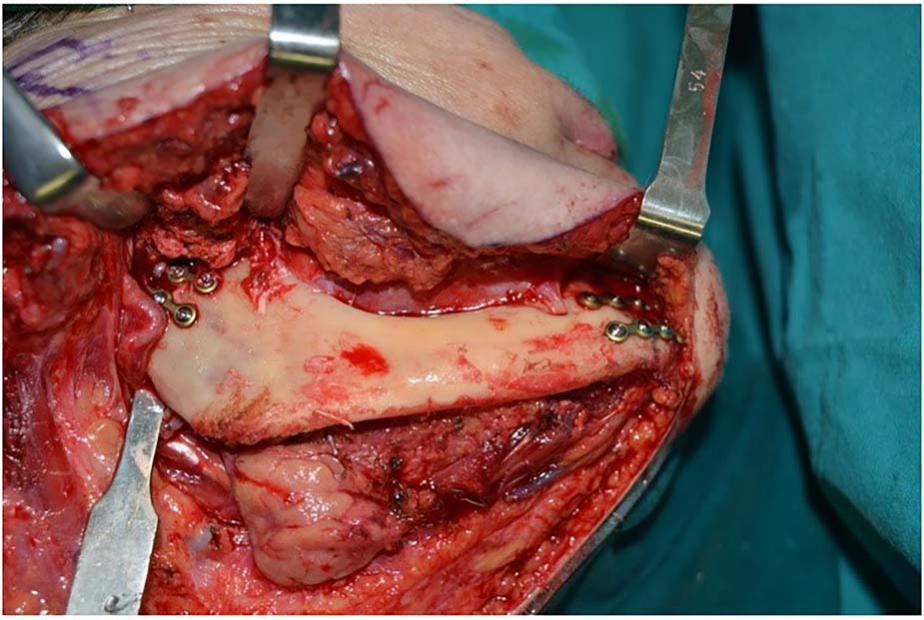
Preplating of two miniplates with four holes, in the parasymphysis and in the subcondylar area.

**Figure 4:**
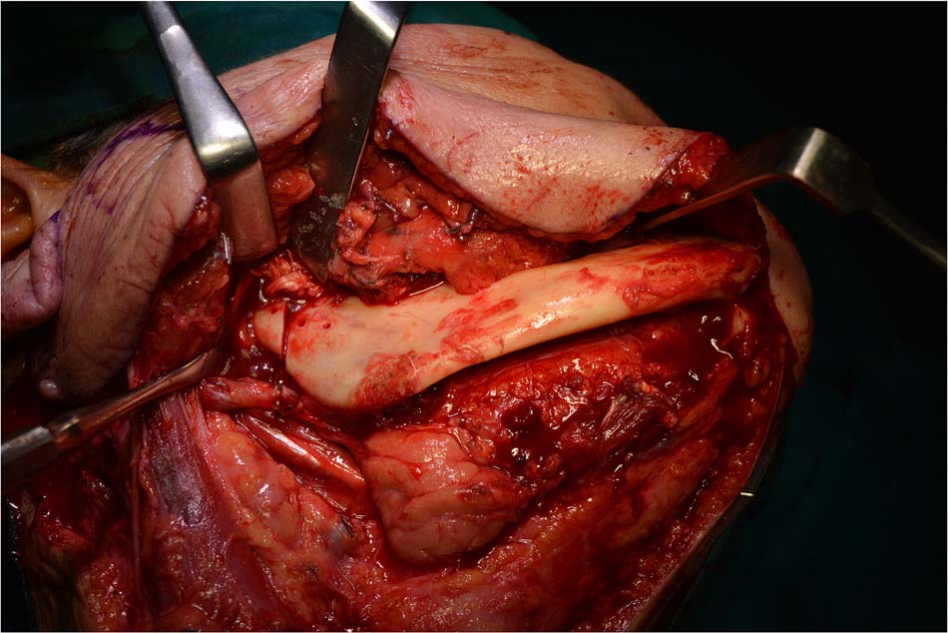
Osteotomy in the parasymphysis and the subcondylar area, after removal of the preplated miniplates.

**Figure 5:**
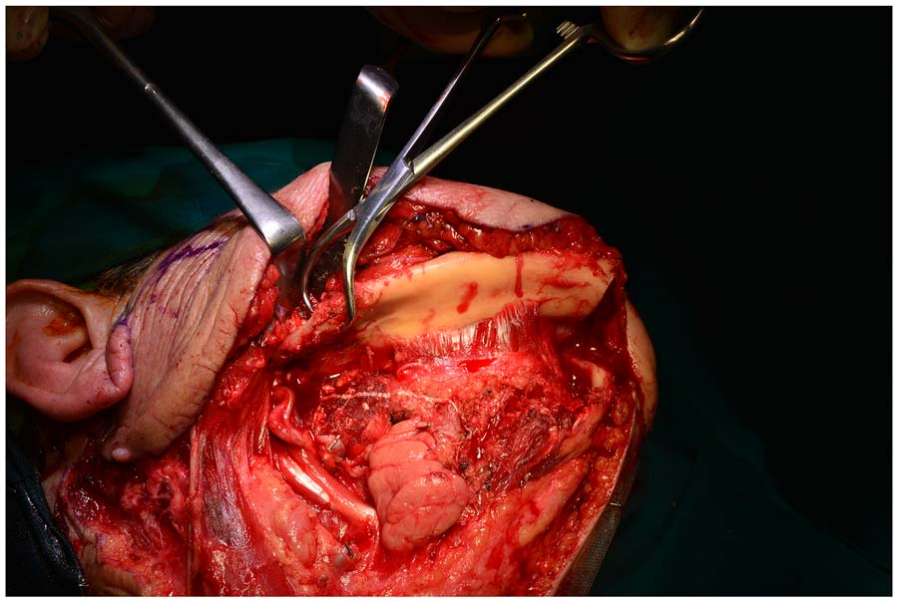
The mandibular ‘swing’.

**Figure 6:**
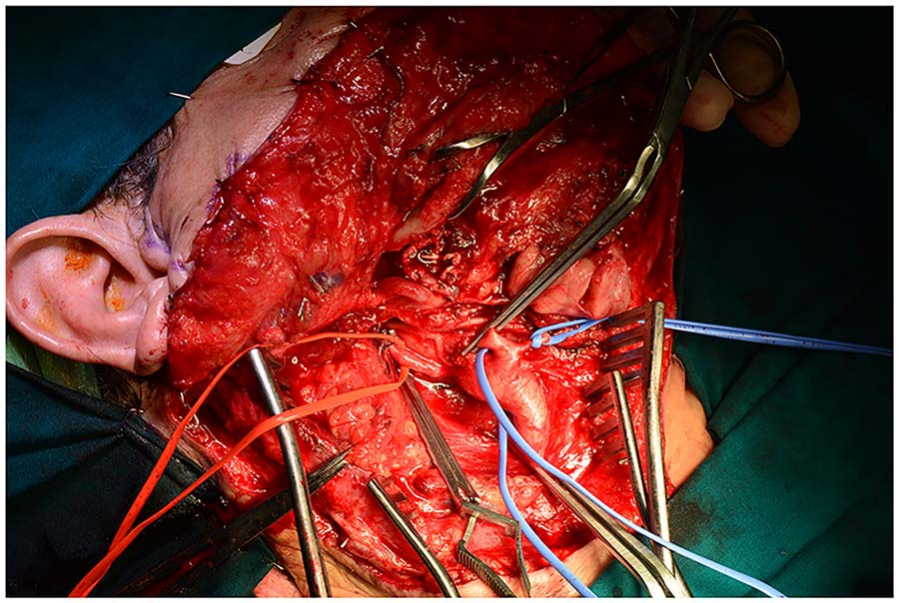
All the branches of the carotid artery are demonstrated prepared, and the aneurysm is demonstrated.

**Figure 7:**
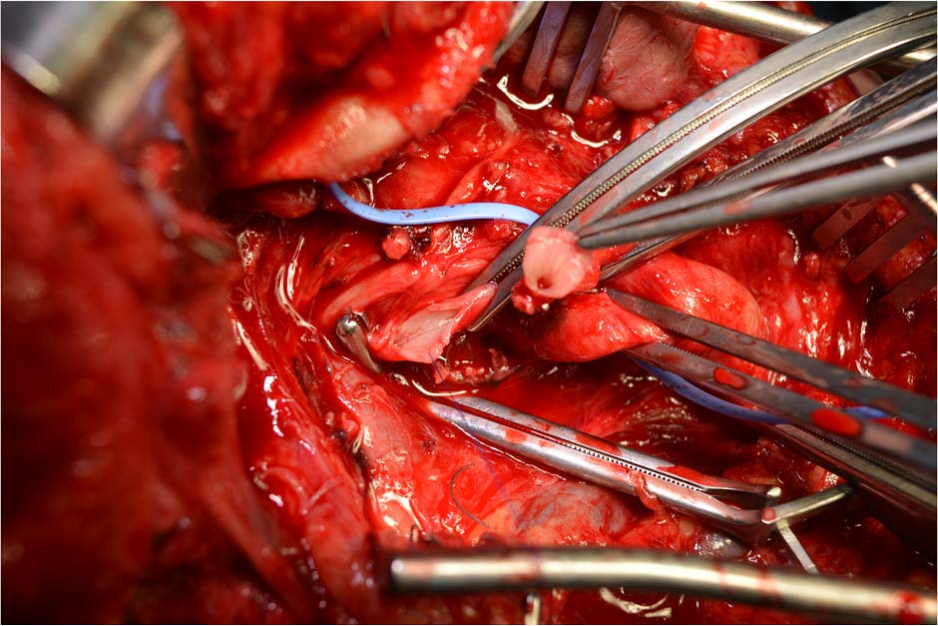
The step before the end-to-end anastomosis of the external carotid artery with the healthy peripheral end of the internal carotid near to the skull base.

**Figure 8:**
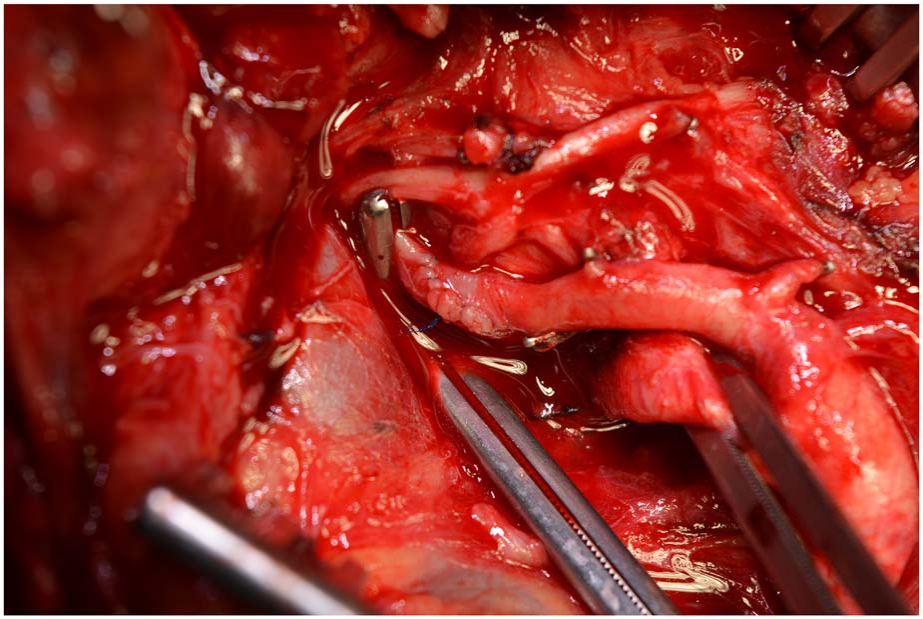
The anastomoses were checked for leakage.

**Figure 9:**
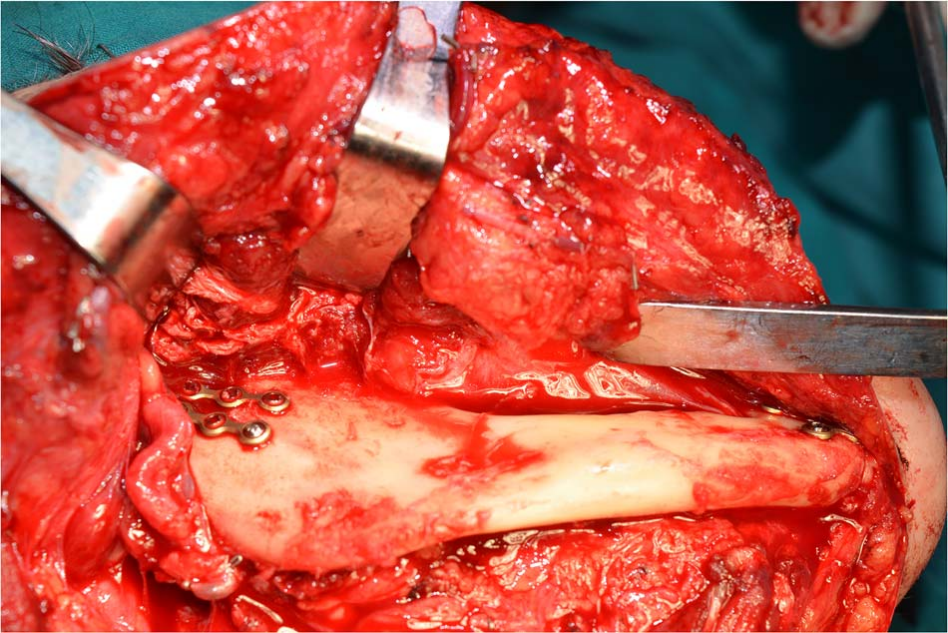
The mandible back in place and the rigid internal fixation.

Operatively, after induction to general anaesthesia via orotracheal intubation, an extended submandibular incision to the right of the neck was performed and the mandible was revealed. The marginal branch of the facial nerve was recognized and protected. Following this, the double osteotomy of the mandible was performed in the parasymphysis—with preplating of two miniplates with four holes—and in the subcondylar area—with preplating of two miniplates with four holes (Figs 3 and 4). The mandibular ‘swing’ that was performed straight after that (Fig. 5) gave space for the dissection of the CCA and the bifurcation. All the branches were prepared, and the aneurysm was accessed (Fig. 6). The reconstruction was achieved by bypassing the aneurysm, trans-positioning of the right ECA and end-to-end anastomosis of the ECA with the healthy peripheral end of the ICA near the skull base, after the lignification of the peripheral end of the ECA and its smaller branches (Fig. 7). The anastomoses were checked for leakage (Fig. 8), the mandible was reduced and the easily fixated rigidly after preplating (Fig. 9).

**Figure 10:**
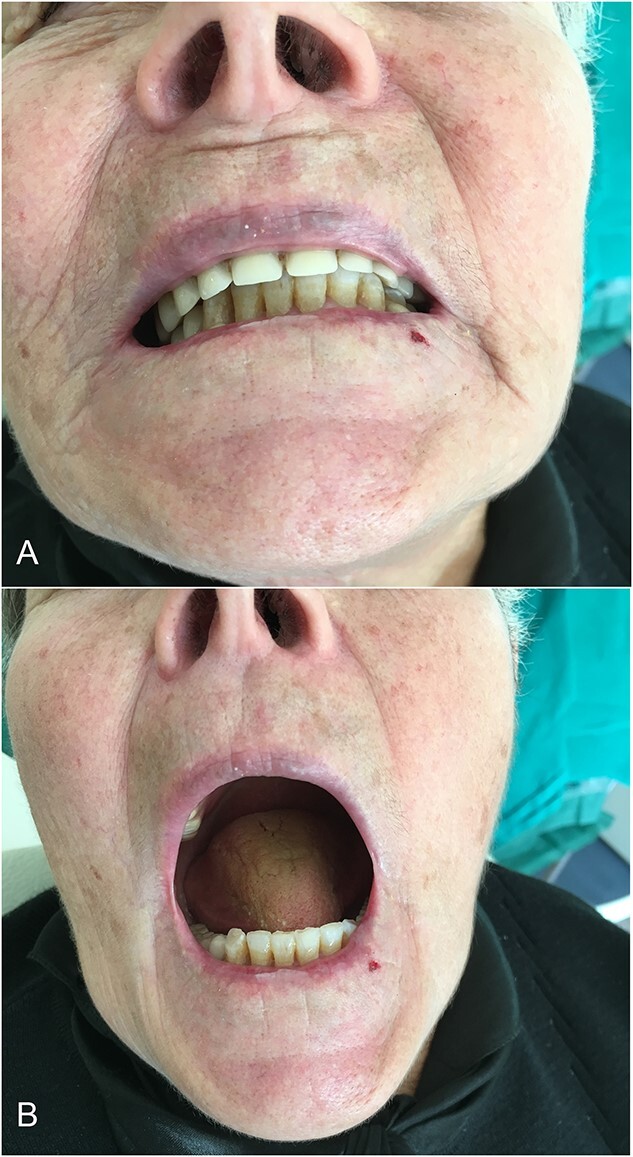
Post-operative mouth opening, and occlusion are normal.

The post-operative period was uneventful (Fig. 10). In the follow-up, the carotid triplex ultrasound showed no abnormal flows. A 3-month post-operative CT angiography showed no aneurysm, and the patient was symptom-free.

## DISCUSSION

ICA aneurysms are defined as 50% or more increase of the arterial diameter [4, 5]. The mean aneurysmatic diameter ranges between 2.35 and 5 cm [8]. The dimensions of the presented aneurysm were 5.5 × 4.5 cm.

Aneurysm symptoms are related to their size, and according to a study, the average diameter of a true aneurysm causing symptoms is 22.8 mm, whereas aneurysms sized 12.9 mm remain asymptomatic [6]. Symptoms may be locally (a palpable mass, hoarseness, dysphagia, Horner’s syndrome) or centrally located (cerebral neurologic events, either transient ischaemic attack or stroke) [9].

According to literature, 42–46% of aneurysms are isolated in the ICA [7, 10]. Mandibulotomy is not described in the aforementioned studies as an approach, which led us to conclude that distal location of aneurysms is rare.

Excluding the simple extracranial carotid artery ligation that carries a considerable risk of stroke and high mortality, aneurysm resection and reconstruction with interposition graft or a patch angioplasty is the preferred method of open surgical treatment, depending on the location and size of the aneurysm, the course of the ICA and its quality [11].

Despite endovascular stent placement being a successful, minimal invasive procedure with low morbidity, the maximum diameter treated with stenting was 26.3 ± 8.9 mm, and none more than 40 mm [12]. Treatment of large aneurysms as the present would be more intricate than smaller ones (Table 1).

**Table 1:** Cases of isolated true ICA aneurysms reported in the literature

Authors/year of publication	Year range of study/Case Report	Carotid aneurysms	True carotid aneurysms/ICA	Size of aneurysm	Location of aneurysm above /below of mandibular angle	Type of surgery applied in relation to the mandible
Schlieve et al. (2017)	1994–2016	1	1		1	DM osteotomy Unknown method of reconstruction
Winterton et al. (2003)	CR	1	1	1.53 cm	Below	End-to-end to the same ICA
Zhou et al. (2006)	1984–2004	42	22	Not provided	Not provided	Not provided
Al Jarrh et al. (2015)	Case Report	1	1	1.68 × 1.3 cm	Below	End-to-end to the same ICA
Fankhauser et al. (2015)	1998–2012	141	25	Mean diam. 22.2 cm	Not provided	Not provided
Garg et al. (2012)	2005–2010	16	4/5	0.6–5 (2.45)	Not provided	Not provided
Jones et al. (2012)	Case Report	1	1/ICA	3 cm	Perimandibular angle	Mandibular subluxation external carotid transposition
Rana et al. (2011)	Case Report	1	1/distal part of ICA	1.4 × 2 cm	Distal ICA	Open surgical clipping
Edwards et al.	Case Report	1	1/proximal ICA	3 × 2.5 cm	Proximal ICA	Vein saphenous graft
Kakisis et al. (2012)	Case Report	1	1/proximal ICA	7 cm	Proximal ICA	End-to-end anastomosis
El -Sabrout et al. (2000)	1960–1995	67	23	2.5–5 cm	3 distal ICA	Not provided

Preoperatively, adequate circulation of the brain is assessed with the Matas test, but other more elective methods have been described, which are not easily accessible, such as angiography-guided balloon occlusion or SPECT–CT [13].

When the anatomy permits the mobilization and transposition of the ECA and anastomosis with the distal part of the ICA is an attractive method [2, 20]. Reconstruction of the carotid artery bloodstream is preferred and can be achieved by a vein or a synthetic graft bypass. In certain cases of ICA elongation, there is a possibility for an end-to-end anastomosis after aneuresmatectomy of the two stumps of the ICA. In our case, despite the elongation of the ICA, the arterial wall quality was corrupted, and this method was infeasible. Nevertheless, in large ICA aneurysms above the Blaisdell line, the simple traction of the mandible is not sufficient to allow such kind of manipulations. The double mandibular osteotomy that initially described as a valuable access for deep lobe parotid gland tumours and paragangliomas may offer a wide surgical field for open ICA operations.

In the present case, the location of aneurysm at the distal part of the ICA and its large dimension were critical for the decision to use the double mandibular operation. The mobilization of the ECA and the anastomosis with the distal stump of the ICA performed easy and quickly after mandibular osteotomy. Post-operatively, there was no complication from the neighbouring with cranial nerves and the lines of osteotomy healed as in mandibular fractures. The sensation of the lower lip remained intact as the lines of osteotomy preserve the integrity of the inferior alveolar nerve.

Concluding, when the aneurysm is located cephalic to the mandibular angle, the ramus is an anatomic obstacle to perform manipulations to the ICA including anastomosis to the distal part of the vessel near the scull base. The double mandibular osteotomy can deliver the ICA from the bifurcation to its entrance to the skull base through the carotid canal and offers an easy, quick, and safe field for anastomosis with minimum post-operative morbidity.
